# Oligomerization of *Clostridium perfringens* Epsilon Toxin Is Dependent upon Caveolins 1 and 2

**DOI:** 10.1371/journal.pone.0046866

**Published:** 2012-10-02

**Authors:** Christine M. Fennessey, Jinsong Sheng, Donald H. Rubin, Mark S. McClain

**Affiliations:** 1 Division of Infectious Disease, Department of Medicine, Vanderbilt University School of Medicine, Nashville, Tennessee, United States of America; 2 Department of Pathology, Microbiology and Immunology, Vanderbilt University School of Medicine, Nashville, Tennessee, United States of America; 3 Research Medicine, VA Tennessee Valley Healthcare System, Nashville, Tennessee, United States of America; Oregon State University, United States of America

## Abstract

Evidence from multiple studies suggests that *Clostridium perfringens* ε-toxin is a pore-forming toxin, assembling into oligomeric complexes in the plasma membrane of sensitive cells. In a previous study, we used gene-trap mutagenesis to identify mammalian factors contributing to toxin activity, including caveolin-2 (*CAV*2). In this study, we demonstrate the importance of caveolin-2 and its interaction partner, caveolin-1 (*CAV1*), in ε-toxin-induced cytotoxicity. Using *CAV2*-specific shRNA in a toxin-sensitive human kidney cell line, ACHN, we confirmed that cells deficient in CAV2 exhibit increased resistance to ε-toxin. Similarly, using *CAV1*-specific shRNA, we demonstrate that cells deficient in CAV1 also exhibit increased resistance to the toxin. Immunoprecipitation of CAV1 and CAV2 from ε-toxin-treated ACHN cells demonstrated interaction of both CAV1 and -2 with the toxin. Furthermore, blue-native PAGE indicated that the toxin and caveolins were components of a 670 kDa protein complex. Although ε-toxin binding was only slightly perturbed in caveolin-deficient cells, oligomerization of the toxin was dramatically reduced in both CAV1- and CAV2-deficient cells. These results indicate that CAV1 and -2 potentiate ε-toxin induced cytotoxicity by promoting toxin oligomerization – an event which is requisite for pore formation and, by extension, cell death.

## Introduction

*Clostridium perfringens* is a gram-positive bacterium prevalent in soil and in the intestinal contents of both humans and animals. The species produces numerous toxins associated with a range of illnesses including food poisoning (normally associated with *C.* *perfringens* enterotoxin, CPE), gas gangrene (associated with α-toxin), and necrotic enteritis (associated with β-toxin). Using a toxinotyping system, strains of *C. perfringens* are categorized into types A through E based on the production of four major lethal toxins: α, β, ε, and ι [1]–[3]. The ε-toxin (produced by types B and D) is the most potent of these toxins and primarily affects livestock in the form of rapidly fatal enterotoxemia. Although human infections by *C. perfringens* type B or D strains are infrequent, evidence does suggest the ε-toxin may be toxic to humans [4]–[7]; the toxin also has been shown to be cytotoxic to cultured human cells [8]–[10]. Because of the potential for toxicity in humans, its extreme potency (ε-toxin exhibits an LD_50_ of approximately 100 ng/kg in mice) and the lack of therapeutics approved for human use, ε-toxin is categorized as a select agent by the U.S. Department of Health and Human Services [11], [12].

Evidence indicates that ε-toxin is a pore-forming protein that causes dysregulated ion homeostasis and cell death. The toxin is hypothesized to bind to a specific receptor on the surface of host cells, localize to cholesterol-rich lipid rafts, and form a heptameric pre-pore followed by insertion of an active pore into the plasma membrane [13]–[15]. The toxin forms an asymmetrical pore allowing the passage of molecules up to 500 Da [16]; this pore is thought to disrupt ion homeostasis and ultimately to result in cell death [17], [18]. Evidence suggests that ε-toxin might bind to a specific glycoprotein receptor on the host cell surface. In previous studies, binding of ε-toxin to both rat brain and mouse kidney was inhibited by proteases or treatments aimed at removing glycosylation [19], [20]. Additionally, we have shown that the highly O-glycosylated membrane-protein hepatitis A virus cellular receptor 1 (HAVCR1) is essential for ε-toxin cytotoxicity and have demonstrated that the toxin binds to the extracellular domain of the protein [8]. However, another recent study suggests that sialidase treatment can enhance toxin binding and cytotoxicity [21].

The interaction between pore-forming toxins and host cells is more complex than simple toxin binding and pore-formation. Studies demonstrate that a broad range of responses are stimulated upon exposure to pore-forming toxins, including signal transduction pathways, lipid and sterol synthesis, the unfolded protein response, caspase-1 activation, and hypoxia repression pathways [22]–[26]. These host cell responses may be protective against low doses of toxin, but the host cell responses may also contribute to cytotoxicity. For example, *Bacillus thuringiensis*’s Cry toxin activates a Mg^2+^-dependent cytotoxic event involving the stimulation of a G protein, adenylyl cyclase, and protein kinase A [27]. Similarly, mitogen-activated/extracellular regulated kinase (MAP2K1, MEK1), protein kinase C (PKC), and calmodulin dependent kinase II (CaMKII) signaling pathways contribute to cell death of eukaryotic cells treated with either BcII toxin from *Bunodosoma caissarum* or EqTxII toxin from *Actinia equine* [28].

Using gene trap mutagenesis in the toxin-susceptible MDCK cell line we recently identified host cell genes that appear to facilitate ε-toxin toxicity [8]. These genes encode a variety of functions including a cell surface receptor (HAVCR1), transcriptional regulation (ZMYND8 and ZBTB20), and signal transduction (BCL3, DUSP5, and CCDC134). We also identified the gene encoding CAV2. CAV2 is a scaffolding protein found associated with CAV1 in cholesterol-rich flask-shaped invaginations (caveolae) in the plasma membrane which are primarily involved in cell signaling and macromolecule endocytosis. In this study, we examine the roles of CAV1 and -2 in ε-toxin-induced cytotoxicity. We demonstrate that cells deficient in CAV1 or -2 exhibit increased resistance to ε-toxin. Furthermore, we show that the toxin interacts with the caveolins, and that caveolins play a role in ε-toxin oligomerization.

## Results

### Gene-specific Knock-down with shRNAs Reveals a Role for CAV2 in ε-toxin-induced Cytotoxicity

**Figure 1:**
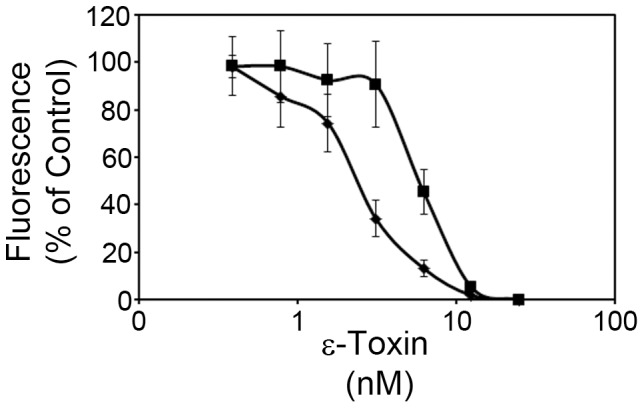
Gene-trap mutagenesis. Wild-type MDCK (⧫) and a *CAV2* mutant cell line (▪) isolated by gene-trap mutagenesis were incubated with serial dilutions of toxin (0–25 nM toxin) at 37°C. Cytotoxicity was determined as described in Materials and Methods. Results were normalized to the fluorescent signal from untreated cells (100%) and cells treated with 1% Triton (0%). Results represent the mean and standard deviation of quadruplicate samples. The mean toxin dose needed to kill 50% of cells was calculated by non-linear regression analysis of results from at least three different experiments (2.7 nM and 6 nM for wild-type and CAV2 mutant cells, respectively). Values were compared by Student's t-test. The *CAV2* mutant cell line required a greater amount of toxin to kill 50% of the cells than was required to kill 50% of the parental MDCK cells (P<0.00005).

In a previous study using MDCK cells, we identified CAV2 as a candidate mammalian gene for ε-toxin-induced cytotoxicity (Figure 1) [8]. In the present study, we sought to confirm a role for CAV2 using an independent cell line, ACHN – a human kidney cell line that is sensitive to ε-toxin [8]. ACHN cells were stably transfected with plasmids expressing gene-specific shRNAs to CAV2 (*CAV2–*53, *CAV2–*56) or GAPDH (used as a negative control).

**Figure 2:**
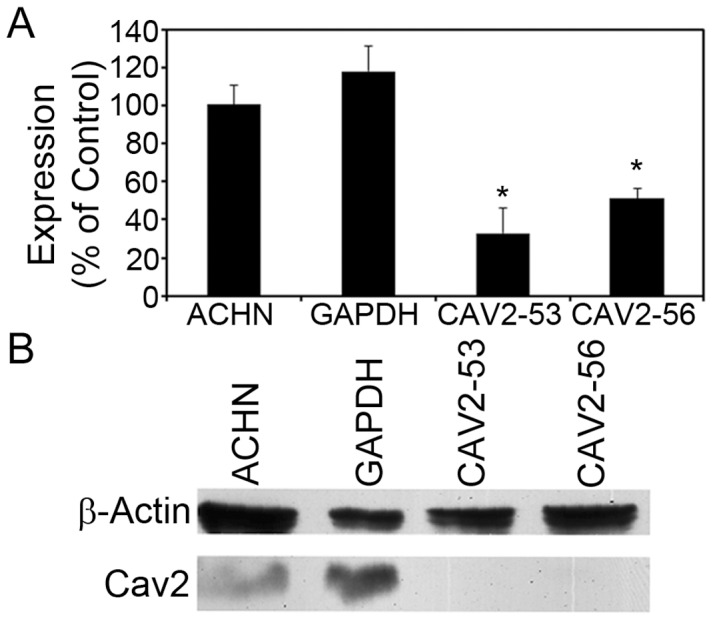
Caveolin 2 expression knockdown by shRNA. A) ACHN cells were stably transfected with gene-specific shRNA to *GAPDH*, or *CAV2* (CAV2*–*53 and CAV2*–*56). Quantitative real-time PCR was used to determine the relative amount of *CAV2* mRNA in transfected and non-transfected ACHN cells. Results of triplicate samples are shown and are expressed relative to non-transfected ACHN cells. The asterisk denotes results that are significantly different from non-transfected ACHN cells (p<0.05, ANOVA followed by Dunnett's post hoc test). B) Whole-cell lysates from non-transfected and stably transfected ACHN cells were immunoblotted with anti-CAV2 antibody; anti-β-actin antibodies were used as a loading control. A representative immunoblot is shown.

To determine whether transfection with gene-specific shRNA resulted in reduced expression of CAV2, we performed quantitative RT-PCR on the transfected cells. A comparison of *CAV2* mRNA levels in non-transfected ACHN cells versus shRNA-transfected ACHN cells demonstrated a significant decrease in *CAV2* mRNA in the CAV2 specific transfections (Figure 2A). As further confirmation that transfection with gene-specific shRNA reduced expression of CAV2, protein extracts were prepared from whole cell lysates of non-transfected and shRNA-transfected cells and subsequently analyzed by immunoblotting. Results indicated a decrease in CAV2 protein expression in *CAV2–*53 and *CAV2–*56 transfectants compared to non-transfected or *GAPDH*-transfected cells (Figure 2B). These results demonstrate that *CAV*2 expression was reduced in cells transfected with *CAV2*-specific shRNA.

**Figure 3:**
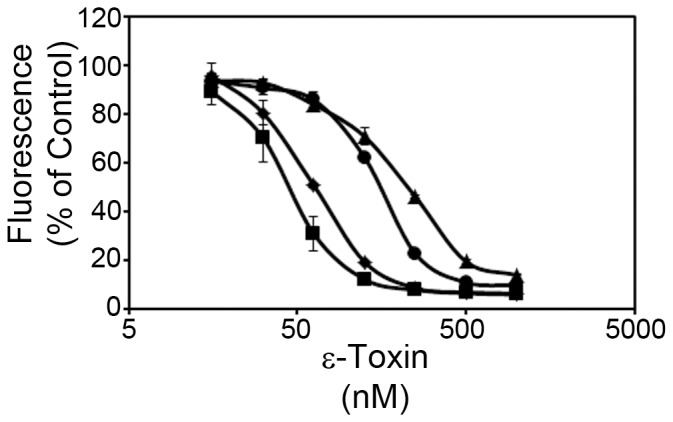
ε-toxin-induced cytotoxicity in caveolin-2-deficient cells. ACHN cells (⧫), or ACHN cells stably transfected with gene-specific shRNA [GAPDH (▪), CAV2*–*53 (•) and CAV2*–*56 (▴)] were incubated with serial dilutions (15.6 to 1000 nM toxin) of purified ε-toxin at 37°C. Cell viability was assessed as described in the Materials and Methods. Results represent the mean and standard deviation of quadruplicate samples. The mean toxin dose needed to kill 50% of cells was calculated by non-linear regression analysis of results from at least three different experiments (60 nM, 55 nM, 158 nM, and 212 nM for wild-type, *GAPDH*-shRNA, CAV2*–*53, and CAV2*–*56, respectively). Values were compared by ANOVA followed by Dunnett's post hoc test. The ACHN cells transfected with *CAV2*-shRNA required a greater amount of toxin to kill 50% of the cells than was required to kill 50% of the parental ACHN cells or cells transfected with *GAPDH*-shRNA (P<0.05). The toxin dose required to kill 50% of cells transfected with *GAPDH-*shRNA was not significantly different than the dose required to kill 50% of wild-type ACHN cells.

We next determined whether decreased expression of CAV2 would result in increased resistance to ε-toxin. Cells were treated with serial dilutions of ε-toxin and cytotoxicity was determined as described in Materials and Methods. Results indicated that CAV2-deficient ACHN cells exhibited increased resistance to ε-toxin (Figure 3) compared to non-transfected or *GAPDH*-transfected cells. Based on these results and the results of the gene-trap selection, we conclude that CAV2 contributes to ε-toxin induced cytotoxicity.

### Caveolin 1 also Contributes to ε-toxin-induced Cytotoxicity

**Figure 4:**
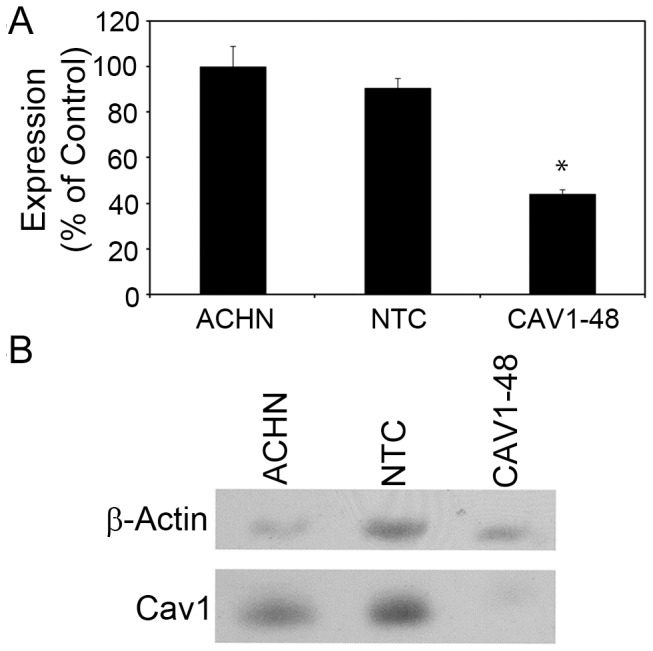
Caveolin 1 expression knockdown by shRNA. A) ACHN cells were stably transfected with a non-targeting control shRNA (NTC) or *CAV1* (CAV1*–*48). Quantitative real-time PCR was used to determine the relative amount of *CAV1* mRNA in transfected and non-transfected ACHN cells. Results of triplicate samples are shown and are expressed relative to non-transfected ACHN cells. The asterisk denotes *CAV1* mRNA levels that are significantly different from non-transfected ACHN cells (p<0.05, ANOVA followed by Dunnett's post hoc test). B) Whole-cell lysates from non-transfected and stably transfected ACHN cells were immunoblotted with anti-CAV1 antibody; anti-β-actin antibodies were used as a loading control. A representative immunoblot is shown.

Caveolin 1 was not identified in our gene-trap selection. However, CAV1 is structurally and functionally related to CAV2. Indeed, CAV2 and CAV1 form heterodimers in the plasma membrane and co-localize in a specialized type of cholesterol- and sphingolipid-rich lipid rafts termed caveolae. Because of this direct interaction, we next sought to determine whether CAV1 is also involved in ε-toxin induced cytotoxicity. To test this hypothesis, ACHN cells were transfected with plasmid DNA expressing shRNA targeting *CAV1* (*CAV1–*48) or a non-targeting control shRNA (NTC). Quantitative RT-PCR of RNA isolated from non-transfected ACHN, and from NTC- and *CAV1–*48-transfected ACHN cells revealed decreased expression of *CAV1* mRNA in cells transfected with *CAV1*-specific shRNA (figure 4A). These results were confirmed by immunoblotting whole cell lysates with anti-CAV1 antibody (Figure 4B). These results demonstrate that caveolin 1 expression was reduced in cells transfected with *CAV1*-specific shRNA.

**Figure 5:**
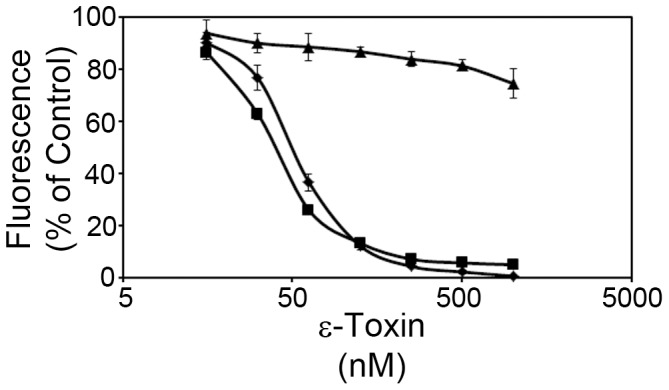
ε-toxin-induced cytotoxicity in caveolin-1-deficient cells. ACHN cells (⧫), ACHN cells stably transfected with a non-targeting control shRNA (NTC) (▪), or ACHN cells stably transfected with *CAV1*-specific shRNA (CAV1*–*48) (▴) were incubated with serial dilutions (15.6 to 1000 nM toxin) of purified ε-toxin at 37°C. Cell viability was assessed as described in the Materials and Methods. Results represent the mean and standard deviation of quadruplicate samples. We did not determine the dose of toxin needed to kill 50% of cells as completing the dose response curve for cells transfected with *CAV1*-shRNA would require considerable amounts of toxin.

To determine whether decreased expression of CAV1 would result in increased resistance to ε-toxin, non-transfected ACHN, and NTC- and *CAV1–*48-transfected ACHN cells were treated with serial dilutions of ε-toxin and cytotoxicity was determined as described in Materials and Methods. Results indicated that CAV1-deficient ACHN cells exhibit increased resistance to ε-toxin (Figure 5). Based on these results, we conclude that CAV1, like CAV2, contributes to ε-toxin-induced cytotoxicity.

### ε-toxin Interacts with Caveolin 1 and Caveolin 2

Caveolins 1 and 2 are widely known for their roles in caveolae-mediated endocytosis. However, it is not clear whether internalization of ε-toxin contributes to cytotoxicity [17], [29]–[31]. Caveolin 1 also has been shown to directly interact with a variety of proteins, including another pore-forming toxin [32]–[38]. Based on these observations, we sought to determine whether caveolin 1 or caveolin 2 interacts with ε-toxin.

**Figure 6:**
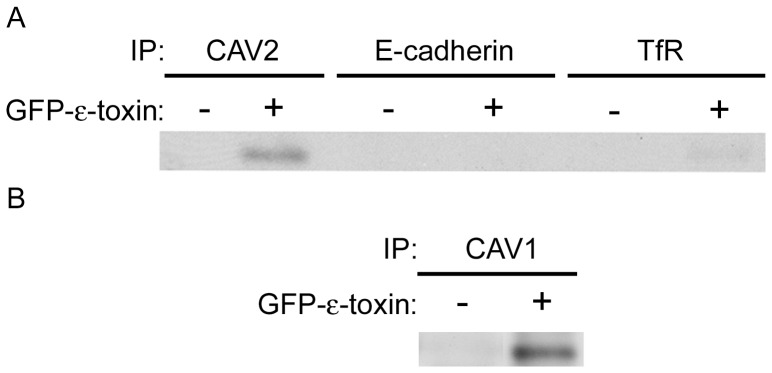
Specific co-immunoprecipitation of ε-toxin **and caveolins.** A) ACHN cells were either untreated (control) or treated with GFP-labeled toxin. Cell lysates were incubated with beads crosslinked to CAV2, E-cadherin or transferrin receptor antibodies. Proteins eluted off the beads were analyzed by immunoblotting with anti-GFP antibody. B) ACHN cells were either untreated (control) or treated with GFP-labeled toxin. Cell lysates were incubated with beads crosslinked to CAV1 antibodies. Proteins eluted off the beads were analyzed by immunoblotting with anti-GFP antibody. Representative immunoblots are shown.

To detect possible interactions between the caveolins and ε-toxin, wild-type ACHN cells were treated with GFP-labeled ε-toxin. GFP-labeled toxin was used instead of wild-type ε-toxin as we do not possess an anti-toxin antibody which recognizes toxin bound to cells. Previous studies demonstrate that GFP-labeled ε-toxin has the same biological effect on cells as unlabeled toxin [19], [39]–[42]. Following incubation of GFP-labeled ε-toxin with cells, cell extracts were prepared and CAV1 or CAV2 was immunoprecipitated as described in Materials and Methods. As controls, immunoprecipitations were performed using antibodies to E-cadherin or transferrin receptor. E-cadherin (like CAV1 and CAV2) localizes to cholesterol-rich membrane microdomains, but unlike CAV1 and CAV2, it is not found in the caveolae domains. Transferrin receptor, though localized to the plasma membrane, is excluded from lipid raft microdomains. Immunoblot analysis of immunoaffinity purified CAV2 or CAV1 demonstrated co-purification of ε-toxin (Figure 6A and B). Results also indicated minimal association of ε-toxin with either E-cadherin or transferrin receptor (Figure 6A). Based on these results, we conclude that ε-toxin physically associates with CAV1 and CAV2 either directly or in a complex and that this association is specific, rather than a coincidence of ε-toxin localization in lipid rafts or of non-specific immunoprecipitation.

**Figure 7:**
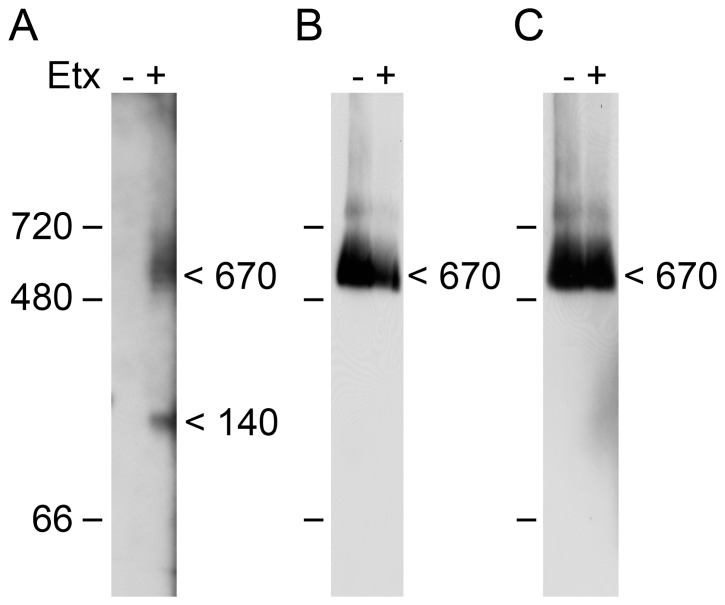
Protein complexes analysed under non-denaturing conditions . ACHN cells were either untreated (control) or treated with GFP-labeled ε-toxin. Membrane protein extracts were separated using blue-native PAGE and analyzed by immunoblotting with anti-GFP (A), anti-CAV1 (B), or anti-CAV2 (C). The positions of proteins molecular weight markers and the estimated sizes of proteins complexes are shown.

To provide additional evidence that ε-toxin and the caveolins co-reside in a multiprotein complex, we analyzed oligomeric complexes formed by the toxin under non-denaturing conditions. A previous study has demonstrated that ε-toxin amasses into a large “mega assemblage” of approximately 670 kDa when added to MDCK cells [43]. Using a similar experimental approach, we observe ε-toxin in protein complexes of approximately 140 and 670 kDa following incubation with ACHN cells (Fig. 7A). Both CAV1 and CAV2 also were observed as part of a 670 kDa complex (Fig. 7B and 7C). The fact that the size of the caveolin-containing complex does not appear to change in the presence of ε-toxin may be an artifact of the limited resolution of this experimental approach and/or the presence of a small proportion of ε-toxin monomers in the complex. Although a more detailed analysis of this complex is required to determine protein stoichiometry as well as the identities of additional proteins present in the complex, these results provide additional evidence supporting our conclusion that ε-toxin and the caveolins colocalize in a multiprotein complex.

### ε-toxin Binding and Oligomerization

**Figure 8:**
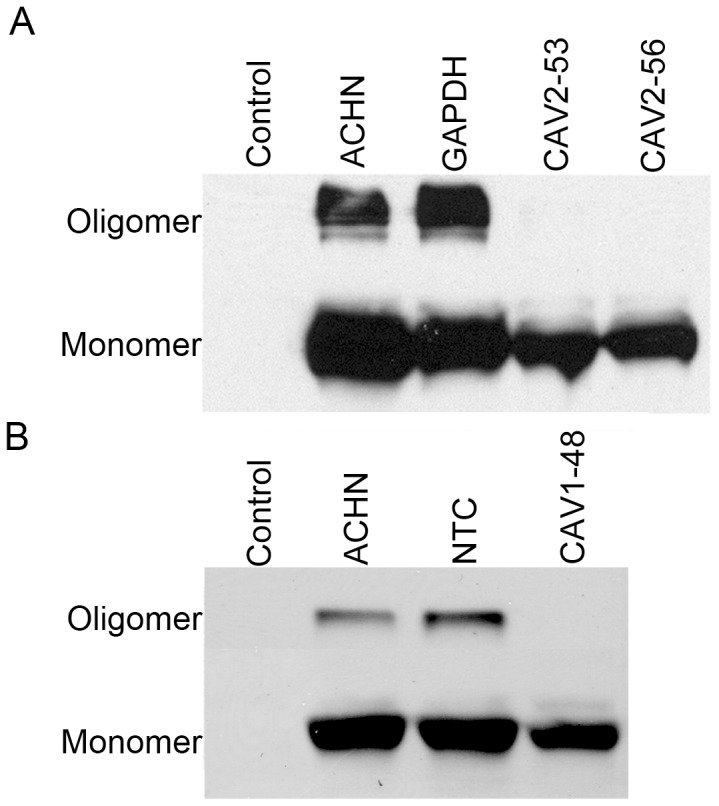
Toxin binding and oligomerization. A) Non-transfected ACHN cells, or cells transfected with shRNA specific for *GAPDH* or *CAV2* were treated with GFP-labeled toxin. Whole cell lysates were analyzed by immunoblotting with GFP antibody. Non-transfected ACHN cells in the absence of toxin served as a control. A representative immunoblot is shown. B) Non-transfected ACHN cells, or cells transfected with a non-targeting shRNA or with *CAV1*-specific shRNA were treated with GFP-labeled toxin. Whole cell lysates were analyzed by immunoblotting with GFP antibody. Non-transfected ACHN cells in the absence of toxin served as a control. A representative immunoblot is shown.

The cytotoxic activity of ε-toxin follows toxin binding and the formation of toxin oligomeric complexes [40], [44]–[46]. To determine whether binding or oligomerization of ε-toxin are disrupted in caveolin-deficient cells, non-transfected ACHN cells or cells transfected with *CAV2*-specific shRNA were treated with GFP-labeled ε-toxin. Cells were subsequently lysed, and toxin binding and oligomerization were assessed by immunoblotting. Results suggest that depletion of CAV2 leads to a modest reduction in the amount of monomeric toxin bound to cells (less than a 2-fold difference, Figure 8A). However, minimal amounts of oligomeric toxin were detected on CAV2 deficient cells (greater than a 60-fold difference compared to the controls, Figure 8A). Similar results were observed in CAV1 deficient cells (greater than an 8-fold difference in oligomeric toxin compared to the controls, Figure 8B). Based on these results, we conclude that CAV1 and CAV2 promote oligomerization of ε-toxin on cells.

## Discussion

Numerous studies suggest that the lethal effect of ε-toxin follows binding of the toxin to a cell surface receptor and the formation of oligomeric pores [15], [17], [40], [44], [45]. However, the specific molecular interactions between ε-toxin and mammalian cells are poorly understood. Results of the present study demonstrate that caveolin-1 and -2 potentiate the cytotoxic activity of ε-toxin towards mammalian cells.

A previous study suggested that CAV2 contributes to ε-toxin-induced cytotoxicity in MDCK cells [8]. In the present study, we have used a human cell line to confirm that cells deficient in CAV2 or its interaction partner CAV1 display increased resistance to ε-toxin. This effect is more dramatic in CAV1-deficient cells than in CAV2-deficient cells, perhaps because CAV2 cannot properly localize to the plasma membrane without the presence of CAV1 [47]; the toxin-resistance observed in CAV1-deficient cells may be the result of reduced levels of both CAV1 and CAV2 in the plasma membrane.

Results from previous studies suggest that ε-toxin interacts with cells through cholesterol- and sphingomyelin-enriched lipid raft domains [31], [48], [49]. Binding and oligomerization of ε-toxin to MDCK cells and to rat synaptosomes was reported to be found exclusively within lipid rafts [48]. Furthermore, single-molecule tracking studies revealed that toxin bound to cells is confined to small (0.4 µm^2^) microdomains on the cell surface and that this confinement was dependent on cholesterol and sphingomyelin [49]. Lipid raft domains also have been implicated in the interactions of numerous other pore-forming toxins including *Staphylococcous aureus* α-toxin and *Aeromonas hydrophila* aerolysin [50]–[56], although the role of lipid rafts in aerolysin activity is conflicting [50], [51], [57]. At least two types of lipid rafts have been identified: planar lipid rafts and caveolae [58], [59]. Planar lipid rafts lack distinguishing morphological features, being in a continuous plane with the plasma membrane. In contrast, caveolae are invaginations of the plasma membrane and contain caveolin proteins. The results of the present study demonstrate that ε-toxin is capable of physically interacting with both CAV1 and CAV2, either directly or as part of a multiprotein complex [43]. The co-immunoprecipitation of ε-toxin with caveolins and evidence indicating that the toxin and caveolins are present in large protein complexes of similar size therefore suggests that the toxin interacts with caveolar lipid-rafts.

Following binding of ε-toxin to cells, the toxin assembles into an oligomeric pre-pore followed by insertion of an ion-conductive pore through the plasma membrane [15], [17], [40], [44], [45]. Formation of the oligomeric complexes is detectable as early as 15 minutes after toxin addition to MDCK cells, at which time 10 to 20% of the monolayer has been killed [17]. Both formation of the oligomeric complexes and cytotoxicity continue to increase with time [17]. Formation of these oligomeric complexes is observed when ε-toxin is added to sensitive, but not resistant, cell lines [45]. In addition, the active form of ε-toxin, but not the inactive prototoxin, is able to form the detergent-resistant complexes [45]. Formation of the toxin oligomers is associated with rapid efflux of intracellular K^+^ and increases in intracellular Ca^2+^, Cl^–^ and Na^+^ [17], [18]. Experiments using artificial lipid bilayers confirmed the channel-forming properties of the toxin, and revealed that pores formed by ε-toxin are approximately 0.5 nm in diameter and allow passage of molecules up to 500 Da in size [16], [18]. However, the ability of the toxin to oligomerize is greatly reduced in caveolin-deficient cells. This reduced capacity of the toxin to oligomerize likely accounts for the observed resistance of caveolin-deficient cells.

Caveolins have been implicated in multiple cellular processes, one or more of which may account for the observed defect in ε-toxin oligomerization [60]. A motif within CAV1, the scaffolding domain, has been shown to interact with a variety of signaling molecules including G-proteins, Src-like kinases, Ha-Ras, and eNOS [32]. Thus, it is possible that the caveolins interact directly with the toxin. Such a model has been proposed to explain the role of CAV1 in promoting the cytotoxicity of *Staphylococcus aureus* α-hemolysin [33]–[38]. However, these results remain controversial as the α-hemolysin motif may not penetrate deep enough into the membrane to interact with CAV1 in the inner leaflet of the membrane [61]. Alternatively, caveolins may interact with a plasma membrane protein whose presence within caveolae contributes to ε-toxin oligomerization. The ε-toxin is believed to interact with a protein receptor or co-receptors (perhaps including HAVCR1) on sensitive cells [8], [17], [20], [62]–[64]. It remains to be determined whether HAVCR1 localizes to caveolae. In addition to participating in protein-protein interactions within membranes, caveolins also have been demonstrated to impact lipid composition and membrane fluidity, though these relationships are complex [60]. For example, both overexpression and depletion of CAV1 from cells may result in increased membrane fluidity [65], [66]. These effects on membrane fluidity are likely the consequence of CAV1 dependent changes to lipid composition of the membrane. For example, cells expressing a dominant-negative CAV1 mutant protein exhibit decreased levels of cholesterol in the plasma membrane [67]. Changes in the levels of membrane cholesterol have been shown to change membrane fluidity [68]–[71] and ε-toxin binding and channel activity (in cholesterol-free artificial membranes) is dependent on membrane fluidity [72]. Additional experiments, beyond the scope of the present study, will be needed to unravel how depletion of the multi-functional caveolins leads to decreased ε-toxin oligomerization.

## Materials and Methods

### ε-toxin Expression and Purification

The gene encoding epsilon prototoxin, *etxB*, from *C. perfringens* type B strain ATCC 3626 was cloned into plasmid pET22b (Novagen). This placed the *etxB* gene under the regulation of the bacteriophage T7 RNA polymerase and fused the C-terminal end of the prototoxin to a His_6_ affinity tag (to aid in purification of the protein). A derivative plasmid that expressed a green-fluorescent protein (GFP)-epsilon toxin fusion protein was also constructed [41].

Expression and purification of recombinant epsilon prototoxin were performed as described previously [40], [73]. Cultures were lysed in Bacterial Protein Extraction Reagent (Pierce), and the lysates filtered through a 0.2 µm filter to remove residual cellular debris. Recombinant ε-prototoxin was purified using Q-sepharose (Pharmacia) followed by Ni-NTA (Qiagen). The eluted prototoxin was concentrated and buffer exchanged using Amicon Ultra 10 kDa centrifugal filter devices with 5 mM tris buffer, pH 7.5. Purified prototoxin was treated with trypsin-bound agarose (Pierce), resulting in pure, active ε-toxin.

Plasmid DNA capable of expressing the -prototoxin (or -toxin) is considered a select agent by the U.S. Department of Health and Human Services.

### Cell Culture

MDCK (Madin Darby Canine Kidney) and ACHN human kidney cell lines were obtained from ATCC. Unless otherwise indicated, cells were cultured in MEM media amended with 10% fetal bovine serum.

To assess cytotoxicity, cells were plated in 384 well dishes at 5000 cells per well in Leibovitz L-15 (supplemented with 10% fetal bovine serum), and serial dilutions of ε-toxin were added to the plate. Cell viability was assessed by addition of resazurin (CellTiter Blue, Promega) to detect metabolically active cells [73]. Fluorescence at 590 nm was measured following excitation at 560 nm using a BioTek FL×800 plate reader. Results were normalized to the fluorescent signal from cells incubated in the absence of toxin (100%) and in 0.1% Triton X-100 (0%).

ACHN cells were transfected with pGIPZ shRNA-expressing plasmids (Open Biosystems V2LHS_112753, V2LHS_112756 and V2LHS_150248) using GenJet In Vitro Transfection Reagent (Version II, SignaGen Laboratories) according to manufacturer’s instructions. In brief, 2 µg shRNA-expressing plasmid DNA was incorporated into GenJet transfection complexes, and applied to 1.2×10^6^ ACHN cells. After a brief incubation at 37°C, cells were plated with complete MEM media in a 6-well dish and incubated for 24 hours. Transfected cells were identified by their capacity to survive in media supplemented with puromycin (2.5 µg/mL), and the cells were subsequently cloned by limiting dilution.

### Reverse Transcription-PCR

RNA was extracted from ACHN cells using TriZol reagent (Invitrogen) followed by treatment with RNeasy Mini Kit and RNAse-free DNAase (Qiagen) according to manufacturer’s instructions. cDNA was prepared from 2 µg RNA using Omniscript reverse transcriptase (Qiagen). Quantitative RT-PCR was conducted using taqman reagents (Applied Biosystems) and gene-specific probes (*CAV1* or *CAV2*) (Applied Biosystems) on an Applied Biosystems StepOne instrument. TFRC (transferrin receptor), TBP (TATA-box binding protein), and RPLP0 (ribosomal protein, large, P0) gene-specific probes (Applied Biosystems) were used as endogenous controls. Results were analyzed as described previously [74].

### Immunoblotting

Wild-type and transfected ACHN cells were lysed with RIPA buffer (50 mM tris, 150 mM NaCl, 0.5% deoxycholate, 0.1% SDS, 1% NP-40; pH 7.8) containing protease inhibitor (EDTA-free Complete Mini, Roche). Proteins were separated by SDS-PAGE, transferred to nitrocellulose, and probed with the appropriate antibody [caveolin 1 (Cell Signaling, catalog # 3238S), caveolin 2 (BD, catalog # 610685), transferrin receptor (TfR) (Invitrogen, catalog # A11130) E-cadherin (CDH1) (AbCam, catalog # ab53033), β-actin (AbCam, catalog ab8227), or GFP (Santa Cruz, catalog # sc-9996)] followed by horseradish peroxidase conjugated secondary antibodies. Supersignal West Femto or Supersignal West Pico (Thermo, Pierce) were used for ECL chemiluminescent detection.

### Immunoprecipitations

Antibodies (caveolin 1, caveolin 2, E-cadherin, or transferrin receptor) were conjugated to M-280 tosyl-activated Dynabeads (Invitrogen) according to manufacturer’s instructions. ACHN cells (5×10^5^ cells/well) were plated in a 6 well dish and incubated at 37°C overnight. Cells were treated with GFP-labeled ε-toxin diluted in prewarmed Leibowitz L-15 media for 30 minutes (unless otherwise specified), washed with PBS (137 mM NaCl, 2.7 mM KCl, 10 mM Na_2_HPO_4_, 1.8 mM KH_2_PO_4_; pH 7.4), and lysed in RIPA buffer (without SDS). Cell lysates were added to prepared Dynabeads, and incubated at 37°C for 30 minutes with shaking. Beads were washed with PBS and the bound proteins subsequently eluted off the beads with 1× Laemelli sample loading buffer (Bio-Rad) (excluding β-mercaptoethanol) and heated to 95°C for 15 minutes. Eluted samples were subsequently analyzed by immunoblotting with anti-GFP or ε-toxin antibody.

### Blue-Native PAGE

ACHN cells (1.25×10^4^ cells/well) were plated in a 24 well dish and incubated at 37°C overnight. Cells were treated with GFP-labeled ε-toxin diluted in prewarmed Leibowitz L-15 media for 30 minutes (unless otherwise specified), washed with PBS, and native membrane proteins were isolated using ProteoExtract Native Membrane Protein Extraction Kit (Calbiochem). Samples then were analyzed by blue-native PAGE [43], [75]–[78]. Protein samples were supplemented with 10% glycerol, 2% n-dodecyl-ß-D-maltoside, and 0.4% Coomassie G-250, applied to a 3.5–16% gradient polyacrylamide gel and electrophoresed under constant voltage at 4°C. After electrophoresis, the gel was incubated in PBS containing 1% SDS for 15 minutes at 25°C. The gel then was heated in PBS containing 1% SDS until the solution began to boil before incubating an additional 10 minutes at 25°C. The gel then was equilibrated in transfer buffer [40 mM Tris, 100 mM glycine, 20% (v/v) methanol and 0.1% SDS] at 25°C for 10 min, and proteins in the gel were transferred to a polyvinylidene difluoride membrane. The proteins on the membrane were fixed in 8% acetic acid for 15 minutes before immunological detection of GFP-labeled ε-toxin, CAV1, or CAV2.

### ε-toxin Oligomerization

ACHN cell monolayers were incubated at 37°C with GFP-tagged ε-toxin diluted in pre-warmed Leibovitz L-15 for 30 minutes (unless otherwise specified). Cells were subsequently washed with PBS and lysed in 1x Laemelli sample loading buffer (Bio-Rad). Lysates were heat inactivated at 95°C for 15 minutes and analyzed by immunoblotting. Quantitative analyses were performed using Quantity One software (Bio-Rad).
